# Epidemiología de la infección por el virus de la hepatitis C en Colombia

**DOI:** 10.26633/RPSP.2021.96

**Published:** 2021-09-16

**Authors:** María C. López-Osorio, Mauricio Beltrán, María-Cristina Navas

**Affiliations:** 1 Facultad de Medicina, Universidad de Antioquia Medellín Colombia Facultad de Medicina, Universidad de Antioquia, Medellín, Colombia.; 2 Organización Panamericana de la Salud Washington D.C. Estados Unidos de América Organización Panamericana de la Salud, Washington D.C., Estados Unidos de América.

**Keywords:** Virus de la hepatitis C, epidemiología, prevalencia, factor de riesgo, Colombia, Hepatitis C virus, epidemiology, prevalence, risk factor, Colombia, Vírus da hepatite C, epidemiologia, prevalencia, fator de risco, Colômbia

## Abstract

**Objetivo.:**

Describir la epidemiología de la infección por el virus de la hepatitis C (VHC) en Colombia.

**Métodos.:**

Revisión crítica de los estudios de epidemiología de la infección por VHC en Colombia mediante búsqueda de artículos originales y revisiones de tema publicados en el período 1989 a 2020 en las bases de datos PubMed, SciELO y ScienceDirect. Además, se revisaron los informes del Instituto Nacional de Salud y de la Cuenta de Alto Costo del Ministerio de Salud y Protección Social.

**Resultados.:**

Los datos de seroprevalencia de anticuerpos anti-VHC en donantes de sangre están en un rango de 1,5% a 0,32%, que corresponden a los informes del inicio y el final del período de estudio, respectivamente. En la población con factores de riesgo se observa una alta prevalencia de infección por VHC, aunque con variaciones a lo largo del tiempo. Con respecto a los genotipos de VHC en Colombia, se han identificado los genotipos 1, 2, 3 y 4 (subgenotipos 1a, 1b, 2a y 3a).

**Conclusiones.:**

En el período de observación, se describe una disminución en la seroprevalencia de la infección por VHC en donantes de sangre y en pacientes en tratamiento con hemodiálisis en Colombia, lo que demuestra el impacto de las políticas de sangre segura y las medidas de bioseguridad. Los estudios en personas que usan drogas ilícitas por vía inyectable indican una alta prevalencia de infección, con diferencias según la región del país. El genotipo 1, subgenotipo 1b, del VHC es el más frecuente en los distintos estudios realizados en Colombia, y el informe más reciente de la Cuenta de Alto Costo del Ministerio de Salud y Protección Social señala que el genotipo 4 es el segundo genotipo más frecuente en el país.

## Abstract (en)

**Objective.:**

To describe the epidemiology of hepatitis C virus (HCV) infection in Colombia.

**Methods.:**

Critical review of epidemiological studies of HCV infection in Colombia. The PubMed, SciELO, and ScienceDirect databases were searched for original articles and reviews on the subject published from 1989 to 2020. Reports from the National Institute of Health and the High Cost Account of the Ministry of Health and Social Protection were also reviewed.

**Results.:**

Data on seroprevalence of HCV antibodies in blood donors range from 1.5% to 0.32%, corresponding to reports at the beginning and end of the study period, respectively. In the population with risk factors, a high prevalence of HCV infection is observed, although with variations over time. With respect to HCV genotypes in Colombia, genotypes 1, 2, 3, and 4 (subtypes 1a, 1b, 2a, and 3a) have been identified.

**Conclusions.:**

In the observation period, a decrease was seen in seroprevalence of HCV infection in blood donors and hemodialysis patients in Colombia, demonstrating the impact of safe blood policies and biosafety measures. Studies in people who inject illicit drugs indicate a high prevalence of infection, with regional differences within the country. HCV genotype 1, subtype 1b, is the most frequent in the different studies carried out in Colombia, and the most recent report of the High Cost Account of the Ministry of Health and Social Protection indicates that genotype 4 is the second most frequent genotype in the country.

## RESUMO

**Objetivo.:**

Descrever a epidemiologia da infecção pelo vírus da hepatite C na Colômbia.

**Métodos.:**

Foi realizada uma revisão crítica de estudos epidemiológicos sobre a infecção pelo HCV na Colômbia por meio de busca de artigos originais e revisões a respeito do tema, publicados no período de 1989 a 2020, nos bancos de dados PubMed, SciELO e ScienceDirect. Também foram analisados os relatórios do Instituto Nacional de Saúde e da Conta de Alto Custo do Ministério da Saúde e Proteção Social.

**Resultados.:**

O índice de soroprevalência de anticorpos anti-HCV em doadores de sangue varia de 1,5% a 0,32%, correspondendo aos relatos do início e do final do período de estudo, respectivamente. Na população com fatores de risco, observa-se prevalência elevada de infecção pelo HCV, embora com variações ao longo do tempo. Em relação aos genótipos do HCV, foram identificados os genótipos 1, 2, 3 e 4 (subgenótipos 1a, 1b, 2a e 3a) na Colômbia.

**Conclusões.:**

No período de interesse, observa-se uma diminuição da soroprevalência da infecção pelo HCV em doadores de sangue e em pacientes em tratamento hemodialítico na Colômbia, o que demonstra o impacto das políticas de sangue seguro e medidas de biossegurança. Estudos com usuários de drogas ilícitas injetáveis indicam alta prevalência de infecção, com diferenças de acordo com a região do país. O subgenótipo 1b do genótipo 1 do HCV é o mais prevalente nos diferentes estudos realizados na Colômbia, enquanto o informe mais recente da Conta de Alto Custo do Ministério da Saúde e Proteção Social indica que o genótipo 4 é o segundo mais frequente no país.

La Organización Mundial de la Salud (OMS) incluyó, en el 2010, las hepatitis virales como una prioridad en los planes de salud pública teniendo en cuenta el peso de la enfermedad (1). La Asamblea Mundial de la Salud estableció que todos los países debían implementar o mejorar los planes de vigilancia epidemiológica y la capacidad diagnóstica de la infección por VHC (2). En el 2016 se presentó la estrategia global del sector salud encaminada a la meta de reducción de 65% de la mortalidad asociada a los virus de las hepatitis para el año 2030 y reducción de 90% en la incidencia. Esta estrategia se fundamenta en el tamizaje de las unidades de sangre, la notificación obligatoria de los casos y el registro de casos de cirrosis y carcinoma hepatocelular, el fortalecimiento de los sistemas de diagnóstico, tratamiento y desarrollo, y la disponibilidad de tratamiento con antivirales (3). La OMS estima 71 millones de personas con hepatitis C crónica y una mortalidad de 400 000 casos al año (1).

El VHC está clasificado en la familia *Flaviviridae,* género *Hepacivirus* (4). A la fecha se han caracterizado 8 genotipos y 90 subgenotipos (4,5). Este virus es agente causal de infección transitoria e infección crónica. La infección transitoria corresponde a los casos con aclaramiento viral espontáneo que corresponden a 15-45% de los pacientes. La infección crónica se desarrolla en 60% a 85% de los pacientes con riesgo de desarrollo de hepatopatías terminales en dos a tres décadas (1). El VHC se transmite principalmente por vía parenteral (por transfusión de sangre y reutilización de agujas y jeringas). Otras vías de contagio, aunque menos frecuentes, son la transmisión sexual y la transmisión vertical; en el caso de la transmisión vertical aumenta el riesgo en recién nacidos de madres con coinfección por VHC y el virus de la inmunodeficiencia humana (VIH). Se ha descrito también la transmisión intrafamiliar (6).

Los hepatocitos son la principal célula blanco del VHC; además, se ha demostrado la infección y la replicación del VHC en linfocitos B, monocitos, macrófagos y células dendríticas las cuales se asocian con patologías extrahepáticas como el linfoma no Hodgkin y la crioglobulinemia, entre otras (7,8).

**FIGURA 1.**
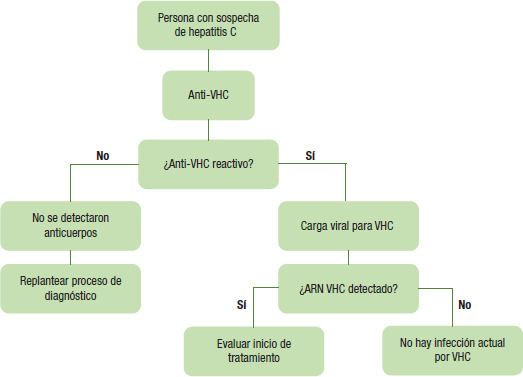
Algoritmo de diagnóstico de la infección por el VHC

En caso de sospecha de infección por VHC se realiza la detección de anticuerpos totales anti-VHC en una muestra de suero del paciente. Los estuches comerciales se basan en el uso de proteínas recombinantes y péptidos sintéticos de las proteínas *core*, NS3, NS4 y NS5 del VHC (9). Por otra parte, están disponibles pruebas rápidas para la detección de anticuerpos anti-VHC en muestras de sangre total y saliva (10). La prueba confirmatoria de infección activa por VHC es la detección del genoma viral (figura 1) (5).

La primera terapia disponible para la hepatitis C crónica fue el interferón alfa de tipo I (IFN alfa); aunque este tratamiento tenía problemas de adherencia, efectos secundarios graves y porcentajes bajos de respuesta virológica sostenida (RVS), se continuó utilizando dado que era la única opción disponible en monoterapia o en combinación con ribavirina. Sin embargo, la pobre respuesta al tratamiento basado en IFN alfa de tipo I en más de 50% de los pacientes con infección por genotipo 1, subgenotipo 1b, representaba un problema y una limitación muy importante en el manejo de los casos. Afortunadamente, el desarrollo de los antivirales de acción directa (AAD) reemplazó la terapia con IFN con buenos resultados, pues permite el aclaramiento viral en más de 95% de los casos (11,12).

***Fuente:*** Guía de práctica clínica para la tamización, diagnóstico y tratamiento de personas con infección por el VHC – Ministerio de Salud y Protección Social, Colombia, 2018.

Con el propósito de aportar al conocimiento y el control de la infección por VHC en Colombia, y teniendo en cuenta que no existe un estudio poblacional a la fecha en el país, se analizaron las publicaciones sobre epidemiología de la infección por VHC en grupos específicos como: donantes de sangre, poblaciones sin factor de riesgo identificado, personas que se inyectan drogas (PID), personas con antecedente de transfusión y personas con otros factores de riesgo.

## MATERIALES Y MÉTODOS

Se realizó una búsqueda no sistemática de literatura científica en las bases de datos PubMed, SciELO y ScienceDirect, buscadores en la red como Google Scholar, en la página de la OMS y en el informe semanal de la Coordinación de la Red de Bancos de Sangre y del Sistema de Vigilancia Epidemiológica del Instituto Nacional de Salud (SIVIGILA). La búsqueda se restringió a las palabras claves (MeSH): “HCV AND Colombia, Hepatitis C Virus AND Colombia, o Hepatitis C AND Colombia” en español e inglés. Además, se realizó una búsqueda en “bola de nieve” a partir de la revisión de referencias citadas en artículos seleccionados y se hizo una revisión de diagnóstico y tratamiento de la infección con énfasis en la guía de práctica clínica para la tamización, el diagnóstico y el tratamiento de la infección por VHC del Ministerio de Salud y Protección Social (13).

Se incluyeron todos los artículos originales de infección por VHC realizados en la población colombiana. Además, se revisaron los informes de notificación del Instituto Nacional de Salud y de la Cuenta de Alto Costo del Ministerio de Salud y Protección Social y las estimaciones realizadas por el Observatorio Polaris del Centro para el Análisis de Enfermedades (CDA, por su sigla en inglés).

Con la estrategia de búsqueda se identificaron inicialmente 2 309 publicaciones que correspondían a artículos completos, mediante la suma de los resultados obtenidos usando las palabras claves en cada base de datos y con redundancia entre las bases de datos, entre las palabras clave o entre ambas. Se seleccionaron 31 artículos en extenso que correspondían a estudios de infección por VHC en la población colombiana. El resto de los artículos fueron excluidos ya que no correspondían a estudios realizados en población colombiana o el objetivo era otra infección diferente a VHC. Alrededor de 76% de los artículos encontrados que cumplían con los criterios de inclusión fueron ubicados mediante las bases de datos PubMed, SciELO, y ScienceDirect, el resto mediante la técnica de búsqueda en bola de nieve.

## RESULTADOS

A la fecha no se han realizado estudios de prevalencia de infección por VHC en la población general en Colombia. Sin embargo, los estudios en donantes de sangre y en poblaciones con y sin factores de riesgo aportan datos importantes para conocer la situación global de la infección por VHC en el país (cuadro 1).

Según los estudios (14-23) y los informes del Instituto Nacional de Salud (INS) (24) publicados entre 1989 y el 2018, la seroprevalencia de infección por VHC en donantes de sangre presenta un rango entre 1,5% y 0,32% de anti-VHC en el período de observación, según el estudio de Robinson y cols. publicado en 1996 (25) y el informe de la Red Nacional de Bancos de Sangre del INS de 2018 (24). El primer estudio corresponde al análisis de 1 033 muestras de donantes obtenidas en cinco bancos de sangre de Medellín en 1989; la prevalencia de anti-VHC fue de 0,97% (10/1 033) (14). Otros estudios realizados entre 1995 y 1997 informan una prevalencia de anticuerpos anti-VHC entre 0,9 y 2,3% en donantes de sangre de diferentes ciudades del país (14–16,25,26).

**FIGURA 2.**
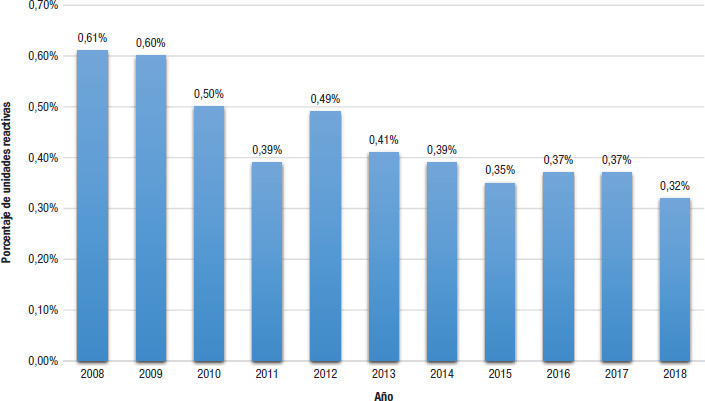
Frecuencia de anticuerpos anti-VHC en donantes de sangre en el periodo 2008-2018 en Colombia

***Fuente:*** Informe Nacional de Indicadores. Coordinación Red Nacional Bancos de Sangre y Servicios de Transfusión, Instituto Nacional de Salud (42)

En estudios realizados después de 1998 se observa una reducción importante en el porcentaje de unidades positivas para anti-VHC. Esta reducción se describe en los informes de la Red Nacional de Bancos de Sangre del INS, en los que se notifica un rango de prevalencia de anti-VHC promedio de 0,61-0,32% de las unidades recolectadas entre 2008 y 2018 (figura 2) (24). Lo mismo se observa en estudios realizados en bancos de sangre en Cali, Tunja, Cúcuta, Medellín y Bogotá, en los que se registra una prevalencia de anti-VHC de 0,56-0,32% en el período entre 2004 y 2018 (cuadro 1) (14-23). Es de destacar que, en los informes de la Red Nacional de Bancos de Sangre del INS, se observan diferencias en la prevalencia de anticuerpos anti-VHC en los donantes de sangre según el departamento. El rango de seroprevalencia es amplio, como se informa en el 2008 (promedio 0,61%; rango entre 2,35% en Caquetá y 0,19% en Sucre), en el 2009 (promedio 0,6%; rango entre 6,5% en Caquetá y 0,11% en Guajira), en el 2010 (promedio 0,5%; rango entre 1,34% en La Guajira y 0,22% en Tolima), y en el 2011 (promedio 0,55%; rango entre 1,09% en César y 0% en Putumayo). En el período 2013-2017 se observa mayor homogeneidad de la seroprevalencia de anti-VHC en la población de donantes de sangre promedio en el país (rango 0,4-0,35%) y también por departamento (24).

Se han realizado estudios en poblaciones sin factores de riesgo, tal como el estudio en 8 130 mujeres (rango de edad: > 20 años a > 75 años) que participaron en el marco de un proyecto de estudio de la infección por el virus del papiloma humano (VPH) en ocho países, incluido Colombia. El estudio permitió, además, estimar la seroprevalencia de infección por VHC en esta población de mujeres. La frecuencia de anticuerpos anti-VHC en el grupo de estudio en Colombia fue de 2,5% (46/1 840; intervalo de confianza de 95% [IC95%]: 1,9-3-3) (27).

**CUADRO 1.** Estudios de prevalencia de la infección por VHC en población colombiana

Población		Período de estudio	Frecuencia de anticuerpos anti-VHC	Detección del genoma viral	Estudio o informe
Donantes de sangre		1989	0,97% (10/1 033)	NA	Echavarría y cols. (1992)
		ND	2,3% (10/430)	NA	Robinson y cols. (1996)
		1995	0,9% (3 328/369 812)	NA	Beltrán y cols. (1997)
		1997	1,5% (15/1 000)	NA	Cortés y cols. (1999)
		1998-1999	0,54% (96/17 895)	NA	Zambrano y cols. (2001)
		2004 - 2005	0,6% (36/6 009)	13,3% (4/30)	Farfán y cols. (2007)
		2007-2010	0,6% (344/54 499)	NA	Bedoya y cols. (2012)
		2012 - 2013	0,5% (104/19 645)	NA	Mejia y cols. (2014)
		2010-2013	0,44%(68/15 461)	NA	Giraldo y cols. (2015)
		2014-2016	0,33% (132/39 825)	13,63% (18/132)	Ruiz y cols. (2018)
		2005-2018	0,567% (833/166 603)	NA	Cardona y cols. (2019)
		2008-2018	0,61% - 0,32%	NA	Red Nacional de Bancos de Sangre (Instituto Nacional de Salud)
Pacientes multitransfundidos	Total	2003	9% (45/500)	26,6% (12/45)	Beltrán y cols. (2005); Di Filippo y cols. (2012)
	Pacientes con tratamiento en hemodiálisis		5/82 (6,1%)	40% (2/5)	
	Pacientes con enfermedades hematooncológicas		8/236 (3,4%)	25% (2/8)	
	Pacientes con sangrado agudo		2/78 (2,56%)	50% (1/2)	
	Pacientes con anemia		1/14 (7,1%)	NA	
	Pacientes con diagnóstico de hemofilia		29/90 (32,25%)	24,1% (7/29)	
Pacientes en tratamiento con hemodiálisis		1989	42,2% (19/45)	NA	Echavarría y cols. (1992)
		2007-2008	2,9% (29/299)	89% (26/29)	Ramírez y cols. (2010)
Pacientes con trasplante de órganos		1989	21,5% (43/200)	NA	Echavarría y cols. (1992)
		2011-2016	6,5% (14/214)	NA	Rojas y cols. (2018)
Pacientes con diagnóstico de hemofilia		1989	6,52 (3/46)	NA	Echavarría y cols. (1992)
		2010-2013	3,7% (6/163)	NA	Prieto y cols. (2014)
		2011-2016	6,5% (14/214)	NA	Rojas y cols. (2018)
Pacientes con coinfección VHC/VIH		2002-2004	0,8% (2/251)	NA	Hoyos y cols. (2006)
		2014	3,15% (29/918)	NA	Toro y cols. (2018)
		2014	3,9% (8/205)	NA	Toro y cols. (2020)
		2011-2016	5,1% (11/214)	NA	Rojas y cols. (2018)
Comunidades indígenas		ND	0% (0/176)	NA	De la Hoz(comunicación personal, 2008)
		ND	5,68% (10/176)	NA	Alvarado y cols. (2011)
Trabajadores sexuales, personal de salud y población desplazada (no discriminados)		ND	2,68% (12/447)	NA	Alvarado y cols. (2011)
Pacientes con diagnóstico de cirrosis, carcinoma hepatocelular o ambos		2005-2007	6,9% (9/131)	44% (4/9)	Cortés- Mancera y cols. (2011)
		ND	0,25% (1/391)	100% (1/1)	Martínez y cols. (2016)
		2009-2012	10% (10/100)	NA	Giraldo y cols. (2014)
		2010-2013	25,3% (19/75)	NA	Prieto y cols. (2014)
		2011-2016	32,6% (70/214)	NA	Rojas y cols. (2018)
Pacientes con transfusión de sangre		2010-2013	62% (101/163)^*^		Prieto y cols. (2014)
		ND	0,25% (1/391)	100% (1/1)	Martínez y cols. (2016)
		2003	6,6% (11/166)	63,6% (7/11)	Arroyave y cols. (2014)
		2011-2016	36,9% (79/214)^*^	NA	Rojas y cols. (2018)
		ND	81,8% (45/55)^*^	100% (45/45)	Yepes y cols. (2016)
Personas que se inyectan drogas		2014	27,3% (251/918)	NA	Toro y cols. (2018)
		2010-2013	1,8% (3/163)	NA	Prieto y cols. (2014)
		ND	31% (82/265)	22,3% (59/265)	Berbesi y cols. (2015)
		2012-2013	22,5 % (16/71)	NA	Sepulveda y cols. (2014)
		ND	17,5% (46/265)	NA	Berbesi y cols. (2017)
		2019	45,54% (92/202)	NA	Cardona-Arias y cols. (2020)
Personas con tatuajes		2011-2016	6% (12/214)	NA	Rojas y cols. (2018)
		ND	9,1% (5/55)	100% (5/5)	Yepes y cols. (2016)
Pacientes con cirugías previas		ND	0,5% (2/391)	100% (2/2)	Martínez y cols. (2016)
		ND	80% (44/55)	100% (44/44)	Yepes y cols. (2016)
Mujeres sin factores de riesgo identificado		1994-2005	2,5% (46/1840)	NA	Quesada y cols. (2015)
Pacientes atendidos en centros dermatológicos		1998	0,01% (2/150)	100% (2/2)	Sanclemente y cols. (2006)
Población general		2019	0,27% (7/2624)	NA	Cardona-Arias y cols. (2020)

VIH, virus de la inmunodeficiencia humana; VHC, virus de la hepatitis C; ND, no disponible; NA, no aplica.

* No corresponden a estudios de prevalencia

Una aproximación a la seroprevalencia población general corresponde a un estudio reciente en 2 624 individuos de 4 ciudades, Bogotá (capital), Medellín (segunda ciudad del país), Pereira (región andina, eje cafetero), Quibdó (Costa Pacífica) y Santiago de Cali (Sur Occidente). La frecuencia de anti-VHC fue de 0,27% (7/2 624) utilizando pruebas rápidas; se destaca que 7,7% de la población de estudio declaró haber recibido transfusiones o trasplantes, aunque no se precisa el año del evento (28).

También se han realizado dos estudios en comunidades indígenas del departamento del Amazonas en los que se han descrito tasas de prevalencia de anticuerpos anti-VHC disímiles, 5,68% (10/184) (29) y 0% (0/176) (comunicación personal Dr. Fernando de la Hoz (30); sin embargo, según la información disponible los estudios no son comparables.

Por otra parte, los estudios en población con factores de riesgo muestran una alta prevalencia de infección por VHC, con variaciones a lo largo del tiempo. El primer informe, en 1989, describe una frecuencia de anti-VHC de 42,2% (19/45) en pacientes en tratamiento con hemodiálisis; 21,5% (43/200) en pacientes que recibieron un trasplante; y 6,52% (3/46) en pacientes con diagnóstico de hemofilia atendidos en Medellín (14). Por otra parte, en pacientes en tratamiento con hemodiálisis en el período 2007-2008 en centros médicos en Santiago de Cali se describe una prevalencia de anti-VHC de 2,9% (29/299) (31).

Un estudio multicéntrico de la Organización Panamericana de la Salud (OPS) realizado en 2003 en hospitales en Bogotá y Medellín permitió el análisis de muestras obtenidas de 500 pacientes con transfusiones múltiples (≥10 unidades de sangre y componentes sanguíneos). La seroprevalencia de anti-VHC fue de 9% (45/500) en la población total de estudio y, según la categoría diagnóstica y el motivo de transfusión, fue de 6,1% (5/82) en pacientes en tratamiento con hemodiálisis; 32,2% (29/90) en casos de hemofilia; 7,1% (1/14) en casos de anemia de células falciformes y talasemia; 3,4% (8/236) en casos de enfermedades oncohematológicas; y 2,56% (2/78) en casos de sangrado agudo. Los factores de riesgo con significancia estadística fueron el diagnóstico de hemofilia (razón de probabilidades [OR, por su sigla en inglés] = 18,03; IC95%: 3,96-114,17), la transfusión de 48 o más unidades de sangre o componentes sanguíneos (OR = 6,08; IC95%: 3,06-12,1) y las transfusiones antes de 1993 (OR = 13,68; IC95%: 6,20-30,86). La prevalencia de infección por VHC mostró una reducción en el grupo de pacientes que recibieron transfusiones entre 1993 y 1995 (7,14%), en comparación con los pacientes que recibieron transfusiones antes de 1993 (27,87%). Incluso la reducción de la prevalencia en el grupo de pacientes que recibieron transfusiones después de 1995 fue de más de 90%, acorde con la cobertura de tamizaje de 100% de unidades de sangre en el país a partir de ese año (32). En un estudio realizado en Medellín se describe un resultado similar en personas con historia transfusional antes de 1994, con una prevalencia de anticuerpos anti-VHC de 6,6% (11/166) (33).

En PID se han realizado varios estudios en diferentes regiones del país con un rango de seroprevalencia entre 7,6% y 47,4% (cuadro 1) (28,33-36). En un importante estudio realizado en 1 123 PID en cinco ciudades del país (Bogotá, Medellín y Cúcuta −en la frontera con Venezuela− y Pereira y Armenia en la región andina-eje cafetero) se describe la infección por VHC. La edad promedio de los participantes del estudio fue de 26,3 ± 6,5 años, y la frecuencia de infección por VHC fue de 28,8 % con un rango entre 7,6 % (Bogotá) y 47,4% (Pereira) según la ciudad. El riesgo de infección por VHC estuvo asociado al uso de drogas inyectables durante cinco años o más (OR = 3,1; IC95%: 1,3-7,2) (35,36). La coinfección de VHC y VIH en esta población fue de 3,2%, con un rango de 1% en Bogotá a 5,6% en Pereira (36). La coinfección de VIH y VHC estuvo asociada con variables como lavado de jeringas y agujas con agua (OR = 3,2; IC95%: 1,6-6,3), traspaso de mezcla de drogas entre jeringas (OR = 2,7; IC95%: 1,3-5,3) y cuatro o más inyecciones por día (OR = 3,5; IC95%: 1,7-7,2), entre otras (36).

Una frecuencia de coinfección de VIH y VHC de 5,1% (11/214) se encontró en pacientes atendidos entre 2011 y 2016 en la unidad de gastrohepatología de la Fundación Valle de Lili, en Santiago de Cali (38); sin embargo, en pacientes con infección por VIH atendidos en centros hospitalarios de la ciudad en Medellín, la coinfección de VIH y VCH fue de 0,8% (2/251) (39).

En un estudio de casos y controles se identificaron factores de riesgo como historia de hospitalización, cirugías o suturas en 55 casos de hepatitis C y 165 controles atendidos en la consulta de gastroenterología y hepatología en ciudades de la costa caribeña (Barranquilla y Cartagena). Ochenta por ciento (44/55) de los pacientes con infección por VHC tenían historia de transfusión sanguínea antes de 1994 (OR = 216; IC95%: 57,7-808) y de 70% a 80% habían sido hospitalizados (OR = 8,9; IC95%: 4,5–17,7) y que habían pasado por cirugías (OR = 5,29; IC95%: 2,55-10,9) o suturas antes de 1994 (OR = 2,8; IC95%: 1,4-5,5) (40).

La infección por VHC también ha sido descrita en otras poblaciones con factores de riesgo en Bogotá, Medellín, Pereira, Quibdó y Santiago de Cali. La mayor prevalencia de infección por VHC fue identificada en personas en situación de calle (2,17%) y en hombres que tienen sexo con hombres (2,09%); mientras que en los trabajadores sexuales (0/380) y en jóvenes vulnerables (0/260) no se detectó ningún caso positivo por prueba rápida para VHC (28).

Aunque se cuenta con datos de seroprevalencia en diferentes poblaciones, los datos de viremia son muy limitados. En algunos estudios realizados en Colombia la amplificación del genoma viral en muestras positivas para anti-VHC, se ha informado un rango entre 13,3% y 100% de positividad en diferentes tipos de población (17,20,30,32,33,39-42). En dos estudios en donantes de sangre se informa la frecuencia de la viremia (13,3% y 13,63%) (17,20).

Los genotipos de VHC circulantes en el país fueron caracterizados por primera vez en 2010 en muestras de donantes de bancos de sangre de Bogotá. El genotipo 1 fue el más prevalente en la población de estudio (88,6%, 31/35); también se identificaron los genotipos 2 (8,6%, 3/35) y 3 (2,9%, 1/35). El subgenotipo 1b se identificó en 82,8% (29/35) de las muestras; y se caracterizaron también los subgenotipos 1a (5,8%, 2/35), 2a (5,8%, 2/35), 2b (2,8%, 1/35) y 3a (2,8%, 1/35). Según el análisis de evolución, se sugiere que el subgenotipo 1b, circula en Bogotá desde 1950, con un crecimiento exponencial desde las década de los 70 y un decrecimiento a principios de los 90, lo que coincide con el inicio del tamizaje obligatorio de las unidades de sangre en el país (44).

Esta distribución de genotipos y subgenotipos es similar a la observada en el estudio de 12 muestras de pacientes con transfusiones múltiples, en el cual se identificó el genotipo 1 en 83,3% de los casos (10/12); (subgenotipo 1b: 66,6%; y subgenotipo 1a: 16,6%); además, se identificaron dos muestras con el patrón de enzimas de restricción (RFLP, por su sigla en inglés) de genotipo 2, subgenotipo 2b (8,3%), y genotipo 3, subgenotipo 3a (8,3%) (41). Se escogieron ocho muestras al azar para secuenciación con el fin de verificar el resultado de genotipificación por la técnica de RFLP. Solo en una muestra se observaron incongruencias con el resultado por RFLP, genotipo 6 y por análisis filogenéticos (genotipo 1, subgenotipo 1b). Esta secuencia presentaba mutaciones ya descritas (41).

Se ha corroborado el predominio del genotipo 1, subgenotipo 1b, del VHC en otros estudios realizados en la población colombiana con antecedentes de transfusiones (33), con diagnóstico de hepatopatías terminales (45) o diagnóstico de infección crónica por VHC (38). También hay un estudio de muestras remitidas a dos laboratorios de referencia (cuadro 2), en el cual se caracterizó el genotipo 1 en el 88,6% de las muestras (1 361/1 538), de las cuales 70% (1 073/1 361) corresponden al subgenotipo 1b; y el 13,5% (209/1 361) al subgenotipo 1a. El genotipo 2 se identificó en 5,4% (83/1 538) y el genotipo 3 en 2% (30/1 538) de las muestras analizadas. Este estudio permitió identificar el genotipo 4 en 62 (4%) de las muestras, lo que corresponde a la primera notificación de la circulación de este genotipo de VHC en Colombia (46).

**CUADRO 2.** Estudios de genotipificación y subgenotipificación del virus de la hepatitis C en Colombia

Genotipo y subgenotipo	Rango de prevalencia (%)
1	2,8-100
1a	5,7-16,6
1b	66,6-80
2	3-8,6
2a	1-5,7
2b	0,4-8,3
3	1,6-8,3
3a	0,5-8,3
4	4-19,2

***Fuente:*** estudios de genotipificación y subgenotipificación publicados en Colombia.

## DISCUSIÓN

El estudio de la epidemiología de la infección por VHC, así como la disponibilidad de datos de mortalidad y morbilidad asociada a esta infección, son recursos clave para establecer la carga de la enfermedad y para el planteamiento de medidas de control y prevención en una población. En Colombia no se conoce la prevalencia de la infección en la población general, lo que podría representar un inconveniente para la toma de decisiones para el control y eliminación de la infección por VHC. Sin embargo, los estudios en donantes de sangre y en la población con y sin factores de riesgo, y el análisis de modelación han contribuido en la estrategia en el país (decreto N° 1692 del 2017) (47).

El Observatorio Polaris del CDA estima 325 600 personas con infección por VHC en Colombia según la predicción realizada con base en la prevalencia de anticuerpos anti-VHC en donantes de sangre de 1,2% (Red Nacional de Bancos de Sangre del INS) y asumiendo una tasa de viremia del 70%, según la historia natural de la infección, lo que corresponde a una prevalencia de anti-VHC de 0,66% en la población. Esta predicción fue fundamental para el Caso de Inversión de Hepatitis C en Colombia liderado por el Ministerio de Salud y Protección Social para evaluar y proyectar la compra centralizada de AAD como parte del compromiso del plan de control y eliminación de las hepatitis virales para el 2030. El esquema de AAD disponible en el país se presenta en el cuadro 3 (13).

Según el SIVIGILA, se notificaron en promedio 228 casos de hepatitis C por año (rango de 185 a 288) en el período 2011-2016 (48). Sin embargo, a partir del año 2017 se observa un aumento significativo con la notificación de 571 casos e incremento continuo en el 2018 (886 casos) y el 2019 (897 casos) (49). Este comportamiento obedece a un avance significativo en la notificación de casos, dado que es requisito para acceder a los AAD adquiridos a través del programa de compra centralizada del Ministerio. Según la Cuenta de Alto Costo, un total de 1 916 pacientes con hepatitis C crónica han recibido tratamiento antiviral entre agosto del 2017 y marzo del 2020. Se informó el aclaramiento viral en 96% de los pacientes en esquema de tratamiento con AAD. El factor de riesgo más frecuente declarado en esta cohorte corresponde a la transfusión sanguínea (25%), seguido por la transmisión sexual (19%) y el equipo de inyección contaminado (2,9%). Es para destacar que, en más de 50% de los casos, no se identificó un factor de riesgo (50).

**CUADRO 3.** Esquema de tratamiento para pacientes con infección crónica por el virus de la hepatitis C

Tratamiento	Duración (semanas)	Alcance (genotipos y subgenotipos)	Dirigido a
Glecaprevir y pibrentasvir	8 a 16	Pangenotípico	Pacientes con o sin experiencia al tratamiento o diagnóstico de cirrosis compensada
Sofosbuvir y velpatasvir	12	Pangenotípico	Pacientes con o sin diagnostico de cirrosis y falla en el tratamiento previo con IFN o inhibidores de NS3
Sofosbuvir + velpatasvir + voxilaprevir	12	Pangenotípico	Pacientes con o sin diagnóstico de cirrosis compensada o falla en el tratamiento previo con inhibidores de NS5A
Ledipasvir y sofosbuvir	8	1a y 1b	Pacientes sin tratamiento previo, carga viral menor a 6 000 000 UI/mL y ausencia de infección por VIH
Ledipasvir y sofosbuvir	12 a 24	1, 4, 5 y 6	Paciente sin tratamiento previo. Puede utilizarse en pacientes con cirrosis compensada. En pacientes con falla previa, el tratamiento dura 24 semanas.
Elbasvir y grazoprevir	12	1a, 1b y 4	Pacientes sin tratamiento previo, diagnóstico de cirrosis compensada y falla en el tratamiento con IFN, carga viral <800 000 UI/mL, en ausencia de pangenotípicos
Paritaprevir y ritonavir + ombitasvir o dasabuvir + ribavirina	12	1a	Pacientes sin tratamiento previo o con falla en el tratamiento con IFN, en ausencia de pangenotípicos
Ombitasvir + paritaprevir o dasabuvir + ritonavir	12	1b	Pacientes sin tratamiento previo o con falla en el tratamiento con IFN, en ausencia de pangenotípicos
Daclatasvir + sofosbuvir	12	1a y 1b	Pacientes con diagnóstico de cirrosis compensada o falla en el tratamiento con IFN e inhibidores de NS3, en ausencia de pangenotípicos

***Fuente:*** elaborado a partir de la Guía de práctica clínica para la tamización, diagnóstico y tratamiento de personas con infección por el virus de la hepatitis C. Ministerio de Salud y Protección Social de Colombia (2019).

IFN, interferón; VIH, virus de la inmunodeficiencia humana.

Por otra parte, los estudios en personas sin factor de riesgo conocido realizados en la población colombiana plantean más interrogantes que respuestas, dadas las limitaciones en la selección y representatividad de la población, además de la ausencia de pruebas confirmatorias en la mayoría de los estudios. Tal es el caso del estudio de la población de mujeres en el marco de un proyecto de VPH. En este estudio se utilizó una prueba doméstica de detección de anti-VHC, utilizando cuatro péptidos de la proteína estructural Core y las proteínas no estructurales NS4 y NS5 del VHC. El ensayo fue validado con estuches comerciales, aunque los autores sugieren la posibilidad de falsos positivos por reacción cruzada con otros flavivirus. Del total de muestras positivas para anti-VHC en el nivel global (405/8130), se analizó 19,5% para la presencia de genoma viral; sin embargo, todas las muestras fueron negativas (27).

Con respecto a los resultados en comunidades indígenas del departamento del Amazonas, es importante tener en cuenta que las transfusiones de sangre o hemoderivados son poco frecuentes en esta población por razones socioculturales (24); en el estudio de Alvarado y cols. se notifica una prevalencia de anti-VHC de 5,68% en indígenas, pero no cuenta con pruebas confirmatorias y, además, no se describen la estrategia ni los criterios de inclusión de los participantes. No es claro si las muestras analizadas fueron obtenidas en el contexto de otro estudio; incluso los resultados de seroprevalencia de otras tres poblaciones presentadas en la publicación de Alvarado y cols. no se discriminan según el factor de riesgo (trabajadores sexuales, personal médico y población desplazada) (cuadro 1) (50). La detección de anticuerpos anti-VHC obtenida en comunidades indígenas podría deberse a falsos positivos por reactividad cruzada notificada en las pruebas serológicas debido a infecciones parasitarias comunes en la población (51), por lo que es indispensable la confirmación del marcador serológico. El otro estudio realizado en indígenas y cols. se encontró una prevalencia de anticuerpos anti-VHC del 0% en esta población. Este estudio se realizó en el marco de un proyecto para evaluar la efectividad de la vacuna recombinante contra VHB en comunidades indígenas y la caracterización de variantes de escape, puesto que el departamento del Amazonas es una región endémica para la infección por VHB (30). En ninguna de las muestras analizadas se detectaron anticuerpos anti-VHC; estas muestras fueron obtenidas de personas mayores de 18 años y positivas para el marcador anti-*core* de VHB (anti-HBc), pertenecientes a comunidades con historia de prevalencia de hepatitis B. Estudios adicionales son necesarios para determinar la prevalencia de VHC en estas y otras comunidades indígenas del país en un tamaño de muestra representativo, así como la identificación de los posibles factores de riesgo.

Los estudios en muestras de donantes de sangre revelan una disminución importante de la prevalencia de anticuerpos anti-VHC de 1,5% a 0,32% a lo largo del tiempo de la ventana de observación 1989-2018 (cuadro 1 y figura 2). Estos resultados demuestran el éxito de las políticas de sangre segura como los criterios de selección de los donantes, las campañas de donación altruista y el impedimento de remuneración a donantes implementados desde 1994 en el país. Un factor importante en la disminución de la prevalencia de anticuerpos anti-VHC en el período de estudio es la selección de donantes y la implementación de cuestionarios más específicos con un impacto positivo en el perfil de los donantes. La resolución N° 00901 emitida por el Ministerio de Salud y Protección Social en 1996 (52) relacionó por primera vez todas las normas técnicas, administrativas y los procedimientos adecuados para bancos de sangre en Colombia; entre ellos, los requisitos para ser donante. Estos requisitos se enfocaron en ocho preguntas: el estado de salud del donante, la edad entre 18 y 65 años, la frecuencia de donación y el antecedente de transfusión, entre otras. Luego se modificó la selección de donantes y se amplió de 8 a 29 preguntas específicas y la definición de las etapas del proceso. El lineamiento técnico para la selección de donantes de sangre en Colombia, emitido por la Red Nacional de Bancos de Sangre en 2013 aclara que el proceso de selección de los donantes debe ser promovido de manera voluntaria (53). Según los informes del INS entre el 2008 y el 2017, el perfil de donante voluntario no repetitivo aumentó de 10,7% a 26,4%, resultado de las campañas de donación realizadas en el país, mientras que la categoría de donante por reposición disminuyó de 28,7% en el 2008 a 5,5% en el 2017 (24). Además, también se debe considerar un posible efecto de cohorte de nacimiento dado que las personas mayores de 65 años no pueden ser donantes, que en parte corresponden a personas nacidas entre 1940 y 1964, generación conocida como los *baby boomers*, que pertenecen a un grupo de riesgo para la infección por VHC en todo el mundo (21,54). Sin duda, la normatividad, los documentos técnicos y las campañas de donación altruista han influido en forma positiva el perfil de los donantes de sangre en el país, lo que se refleja en la disminución de la seroprevalencia de anti-VHC en la población de donantes de sangre y del riesgo residual.

Los datos de detección del genoma de VHC en muestras de donantes de sangre son muy limitados debido a que los lineamientos establecidos por la Red Nacional de Bancos de Sangre exigen únicamente tamizaje de anticuerpos anti-VHC sin prueba confirmatoria de detección del genoma. Los dos únicos estudios en donantes de sangre informan un porcentaje de detección del genoma de 13,3% y 13,63% (17,20). Por otra parte, la frecuencia de detección del genoma viral en estudios de población con factores de riesgo presenta un rango entre 22,3% y 100% en muestras positivas para anti-VHC. Los porcentajes modestos de amplificación del genoma en algunos estudios podría deberse a la carga viral baja y al tiempo prolongado y las condiciones de almacenamiento de las muestras de suero y plasma, como fue descrito por di Filippo y cols. (41); además de la eficiencia de la técnica de extracción de ácido ribonucleico (ARN) (33), dado que se ha demostrado que la probabilidad de amplificación por reacción de cadena de la polimerasa (PCR) del genoma viral se asocia con cargas virales mayores a 100 copias/mL (55).

Evidentemente, la reglamentación e implementación de las normas de bioseguridad en los hospitales y centros de salud del país ha permitido la disminución de la frecuencia y riesgo de infecciones iatrogénicas (40). El estudio de casos y controles aporta evidencia del riesgo de infección por VHC en pacientes hospitalizados, que pasaron por cirugías o suturas antes de 1994 en centros hospitalarios de la región caribe. Estos resultados sugieren una transmisión intrahospitalaria probablemente asociada a malas prácticas clínicas, y plantean la posibilidad de problemas en centros de atención en salud en hospitales de esta y otras regiones del país, antes de la implementación del tamizaje de unidades de sangre, así como de la normativa de bioseguridad.

La reducción en la prevalencia de la infección por VHC en pacientes sometidos a hemodiálisis según lo observado en el primer estudio en 1989 (14) y los estudios posteriores (31,32,56) sugieren que la transmisión del VHC en estas unidades ha disminuido de manera significativa gracias a las normas de bioseguridad y al seguimiento de los pacientes para identificar los casos positivos para VHC; esta estrategia permite la asignación de equipos de hemodiálisis a los pacientes positivos para este u otros virus de transmisión parenteral (56).

Con respecto a la prevalencia de infección por VHC en poblaciones con factores de riesgo, las PID presentan la mayor prevalencia en el país según los estudios más recientes, en particular en ciudades del eje cafetero. Este resultado coincide con lo descrito en países industrializados, donde son el principal grupo de riesgo (5). El manejo de la infección por VHC en esta población exige una intervención que asegure las condiciones para el acceso al diagnóstico, el tratamiento y el seguimiento de los casos; además de las campañas de promoción y prevención que permitan la atención de la condición de adicción, la no reutilización de agujas y jeringas y, por lo tanto, a la disminución del riesgo de infección por VHC.

El rango de coinfección de VIH y VHC (0,8%-5,1%) descrito en los diferentes estudios en el país es menor cuando se lo compara con la frecuencia de coinfección observada en otros países de la región (38,57-60). Es así como en el estudio realizado en Edmonton, Canadá se informa una coinfección de VIH y VHC de 22,8% en PID (58). Otro estudio realizado en Vancouver demuestra coinfección de VIH y VHC en 16% de 1 400 personas que se inyectan (60).

En Brasil se ha descrito una prevalencia de coinfección de VIH y VHC de 6,9% (59/848) y 9,7% (48/495) en dos estudios de PID; como factores de riesgo se identificó la reutilización de agujas y jeringas, historia de transfusión antes de 1994 y tener múltiples parejas sexuales (61,62). En Argentina se han hecho estudios de prevalencia de anti-VHC en PID, trabajadores sexuales y en hombres que tienen sexo con hombres, que notifican una frecuencia entre 1,9% (13/694) y 4,3% (26/602) (63,64). Mientras, en México, la infección por VHC en reclusos usuarios de drogas por vía inyectable presenta una seroprevalencia de 3,3% (129/3 910) (65).

Los resultados de coinfección de VIH y VHC obtenidos en Colombia pueden estar relacionados con el tiempo de cuatro años o menos de historia de uso de drogas vía inyectable y una edad 25 años o menos de las PID. Los autores sugieren que, en Colombia, las PID pertenecen a la población joven y, por lo tanto, representan una oportunidad de intervención (36). Además, estos datos sugieren un peso diferencial en los factores de riesgo asociados a la infección por VHC y la infección por VIH en Colombia cuando se la compara con otras poblaciones; y plantea la importancia de realizar una mejor caracterización de los grupos de riesgo y el seguimiento, dado que hay condiciones dinámicas como el acceso a drogas inyectables.

Los estudios señalan una diferencia importante de la prevalencia de anticuerpos anti-VHC en PID en Bogotá (7,6%) en comparación con la observada en PID en Pereira (43,1%). Esta diferencia puede ser consecuencia del perfil de PID que participaron en el estudio en cada ciudad. En Bogotá, la población PID corresponde a estudiantes universitarios, que informan, además de drogas psicoactivas inyectadas, el consumo de drogas fumadas e inhaladas. Por otro lado, en Pereira, la PID del estudio corresponden a personas en situación de calle, en quienes la reutilización de agujas y jeringas es muy frecuente. Por ende, los autores sugieren que las diferencias estarían a asociadas al nivel de educación y a los recursos económicos disponibles para el recambio de las jeringas según el perfil de PID en cada ciudad (36).

El predominio del genotipo 1, subgenotipo 1b, en donantes de sangre y con historia transfusional en el país, coincide con lo descrito en estudios en diferentes países latinoamericanos y europeos (44). En el estudio de pacientes con transfusiones múltiples el genotipo 1, subgenotipos 1a y 1b, fue descrito exclusivamente en pacientes con diagnóstico de hemofilia transfundidos entre 1950 y 1980, lo que sugiere la asociación de la infección con la transfusión de componentes sanguíneos importados y coincide con el análisis de evolución descrito por Alvarado y cols. (44); mientras que los genotipos 2 y 3 se identificaron en pacientes en tratamiento con hemodiálisis y pacientes con diagnóstico de enfermedad oncológica, respectivamente, además del antecedente de transfusión después de 1998 (41). Mora y cols. describen en la población donante de sangre una de las prevalencias más altas notificadas para el genotipo 1 en todo el mundo (82,8%); además, estimaron que el genotipo 1 de VHC, subgenotipo 1b, fue introducido en Bogotá en 1950 y tuvo un crecimiento exponencial en la transmisión entre 1970 y 1990, posiblemente asociado a la importación de componentes sanguíneos al país (44).

La caracterización de genotipos ha sido de gran importancia en el diagnóstico y el tratamiento de la infección por VHC, teniendo en cuenta el requisito de la genotipificación para la selección del esquema de tratamiento basado en el IFN alfa de tipo I y la probabilidad de resistencia en casos de infección por VHC genotipo 1, subgenotipo 1b (66,67). Con el desarrollo y disponibilidad de los AAD, no es necesaria la identificación del genotipo, dado que se cuentan con antivirales pangenotípicos (67,68). No obstante, la genotipificación sigue siendo de utilidad para determinar su distribución geográfica y caracterizar los genotipos prevalentes por grupos de riesgo, lo que se podría considerar como un marcador epidemiológico que permita rastrear el origen de la infección por VHC y la introducción de nuevos genotipos a la población, como es el caso de genotipo 4 en Colombia (67); tal y como se describe en el más reciente informe de la Cuenta de alto costo con un incremento muy importante en la frecuencia del genotipo 4 que lo sitúa como el segundo genotipo más frecuente después del genotipo 1, subgenotipo 1b, en el país (50). Este resultado señala la necesidad de realizar estudios para caracterizar los factores de riesgo de estos pacientes y documentar la introducción de este genotipo prevalente en Egipto. Además, la genotipificación es indispensable en casos de VHC con factores de riesgo, dada la posibilidad de reinfección luego del tratamiento con AAD (68,69).

Esta revisión permitió describir aspectos clave de la epidemiología del VHC en Colombia, incluida la epidemiología molecular, como una fuente de información para la toma de decisiones en el país. Se revisaron y se tuvieron en cuenta todos los artículos que informaron una prevalencia de infección por VHC en Colombia para la generación de nuevo conocimiento, la cual apoya la estrategia de control y eliminación de las hepatitis virales establecida por la OMS. No obstante, el alcance de este manuscrito tiene limitaciones por la ausencia de estudios poblacionales en Colombia, la falta de datos de viremia en los estudios realizados, dado que la mayoría de los estudios corresponden a análisis de seroprevalencia y por las condiciones técnicas que pudieron interferir en la detección del genoma viral. Por último, se debe considerar que se realizó una revisión crítica de las publicaciones y no una revisión sistemática.

### Conclusión

Las políticas de sangre segura han reducido de manera importante el riesgo de transmisión del VHC por transfusión en Colombia. Se constata la reducción en la seroprevalencia de anti-VHC en varios grupos poblacionales a lo largo del tiempo como donantes de sangre y pacientes en tratamiento con hemodiálisis. Sin embargo, otras vías de transmisión, como la reutilización de agujas y jeringas, está tomando relevancia en nuestro país, como se demuestra en la alta prevalencia de infección por VHC en PID en diferentes regiones de Colombia. La frecuencia de anti-VHC observada en comunidades indígenas y en población sin factores de riesgo sugiere la necesidad de nuevos estudios y el desarrollo de estrategias para el control y prevención de la infección por VHC en Colombia. Sin duda, un estudio a gran escala en la población general sería de gran valor para completar el panorama de la epidemiología de la infección por VHC en Colombia.

## Declaración.

Las opiniones expresadas en este manuscrito son responsabilidad del autor y no reflejan necesariamente los criterios ni la política de la *RPSP/PAJPH* y/o de la OPS.
